# Long Isoforms of NRF1 Contribute to Arsenic-Induced Antioxidant Response in Human Keratinocytes

**DOI:** 10.1289/ehp.1002304

**Published:** 2010-08-30

**Authors:** Rui Zhao, Yongyong Hou, Peng Xue, Courtney G. Woods, Jingqi Fu, Bo Feng, Dawei Guan, Guifan Sun, Jefferson Y. Chan, Michael P. Waalkes, Melvin E. Andersen, Jingbo Pi

**Affiliations:** 1 School of Forensic Medicine, China Medical University, Shenyang, China; 2 Hamner Institutes for Health Sciences, Research Triangle Park, North Carolina, USA; 3 School of Public Health and; 4 First Clinical College, China Medical University, Shenyang, China; 5 Department of Laboratory Medicine and Pathology, University of California–Irvine, Irvine, California, USA; 6 National Toxicology Program, National Institute of Environmental Health Sciences, National Institutes of Health, Department of Health and Human Services, Research Triangle Park, North Carolina, USA

**Keywords:** apoptosis, arsenic, cytotoxicity, KEAP1, keratinocyte, NRF1, NRF2, oxidative stress

## Abstract

**Background:**

Human exposure to inorganic arsenic (iAs), a potent oxidative stressor, causes various dermal disorders, including hyperkeratosis and skin cancer. Nuclear factor–erythroid 2–related factor 1 (NRF1, also called NFE2L1) plays a critical role in regulating the expression of many antioxidant response element (ARE)-dependent genes.

**Objectives:**

We investigated the role of NRF1 in arsenic-induced antioxidant response and cytotoxicity in human keratinocytes.

**Results:**

In cultured human keratinocyte HaCaT cells, inorganic arsenite (iAs^3+^) enhanced the protein accumulation of long isoforms (120–140 kDa) of NRF1 in a dose- and time-dependent fashion. These isoforms accumulated mainly in the nuclei of HaCaT cells. Selective deficiency of *NRF1* by lentiviral short-hairpin RNAs in HaCaT cells [*NRF1*-knockdown (KD)] led to decreased expression of γ-glutamate cysteine ligase catalytic subunit (GCLC) and regulatory subunit (GCLM) and a reduced level of intracellular glutathione. In response to acute iAs^3+^ exposure, induction of some ARE-dependent genes, including NAD(P)H:quinone oxidoreductase 1 (*NQO1*), *GCLC*, and *GCLM*, was significantly attenuated in *NRF1*-KD cells. However, the iAs^3^-induced expression of heme oxygenase 1 (*HMOX-1*) was unaltered by silencing *NRF1*, suggesting that HMOX-1 is not regulated by NRF1. In addition, the lack of NRF1 in HaCaT cells did not disturb iAs^3+^-induced NRF2 accumulation but noticeably decreased Kelch-like ECH-associated protein 1 (KEAP1) levels under basal and iAs^3+^-exposed conditions, suggesting a potential interaction between NRF1 and KEAP1. Consistent with the critical role of NRF1 in the transcriptional regulation of some ARE-bearing genes, knockdown of *NRF1* significantly increased iAs^3+^-induced cytotoxicity and apoptosis.

**Conclusions:**

Here, we demonstrate for the first time that long isoforms of NRF1 contribute to arsenic-induced antioxidant response in human keratinocytes and protect the cells from acute arsenic cytotoxicity.

Chronic exposure to high levels of inorganic arsenic (iAs) is associated with a wide range of human ailments, including cancer, arteriosclerosis, hypertension, type 2 diabetes, and a variety of skin disorders (Pi et al. 2000; Yoshida et al. 2004). The skin is one of the organs most sensitive to iAs toxicity. This is potentially due to the high affinity of arsenic for sulfhydryl groups, which leads to arsenic accumulation and retention in keratin-rich skin tissue. Arsenic-induced nonmalignant skin lesions, including hyperkeratosis and pigmentation disorders, are some of the most common and earliest signs of chronic iAs exposure (Pi et al. 2000; Yoshida et al. 2004). The proliferative skin lesions associated with human iAs exposure include Bowen’s disease and squamous cell or basal cell carcinoma [International Agency for Research on Cancer (IARC) 1987; Wong et al. 1998]. Although iAs is a confirmed human skin toxicant, the underlying molecular mechanism(s) is still unclear. Accumulating evidence suggests that oxidative stress occurs in response to iAs exposure (Pi et al. 2002, 2003) and may be one important factor in dermal arsenic toxicity, including carcinogenesis. Indeed, evidence of arsenic-induced oxidative DNA damage has been observed in cell-based systems (Kojima et al. 2009; Pi et al. 2005) and in the biological samples of rodents and humans (Piao et al. 2005; Yamauchi et al. 2004).

The nuclear factor–erythroid-2–related factors (NRFs) belong to the cap‘n’collar (CNC) subfamily of basic-region leucine zipper (bZIP) transcription factors, which include NRF1 (NFE2L1/LCRF1/TCF11), NRF2 (NFE2L2), NRF3 (NFE2L3), and the nuclear factor–erythroid 2 p45 subunit, as well as more distantly related factors such as BTB and CNC homology 1 (BACH1) and BACH2 proteins (Motohashi et al. 2002). Both NRF1 and NRF2 form heterodimers with small Maf or other bZIP proteins and bind to *cis*-acting element(s) termed antioxidant or electrophile response elements (AREs; also known as EpREs) in the proximal promoters of target genes (Motohashi et al. 2002), leading to activation of transcription (Biswas and Chan 2010; Venugopal and Jaiswal 1998). Although NRF3 can heterodimerize with MafK or MafG and bind AREs (Chenais et al. 2005; Kobayashi et al. 1999), the role of NRF3 in the regulation of ARE-responsive genes remains elusive. NRF1 (Wang and Chan 2006) and NRF3 (Nouhi et al. 2007; Zhang et al. 2009a) are targeted to the endoplasmic reticulum (ER), whereas NRF2 is localized primarily to the nucleoplasm and cytoplasm. Supporting the importance of NRF1 in the developmental process is the finding that loss of NRF1 function in mice results in late-gestational embryonic lethality (Chan et al. 1998). Liver-specific disruption of *Nrf1* results in the development of steatohepatitis and hepatic neoplasms (Xu et al. 2005). In contrast, *Nrf2*-deficient mice are viable but show a higher susceptibility to both oxidative damage and chemical carcinogenesis (Chan and Kan 1999; Chan et al. 2001; Ramos-Gomez et al. 2001), whereas *Nrf3*-null mice develop normally and reveal no obvious phenotype (Derjuga et al. 2004). Fibroblasts derived from *Nrf1*-mutant embryos showed decreased glutathione (GSH) levels and enhanced sensitivity to the toxic effects of oxidants (Chan and Kwong 2000; Kwong et al. 1999), suggesting critical roles for NRF1 in cellular oxidative defense.

Previous studies (Aono et al. 2003; Du et al. 2008), including our own (Pi et al. 2003; Fu et al. 2010), have demonstrated that NRF2 is a key player in the cellular adaptive response to inorganic arsenite (iAs^3+^)-induced oxidative stress. In contrast, the role of NRF1 in arsenic-induced antioxidant response and cytotoxicity has not been established. In the present study, we examined the distinctive roles of NRF1 in iAs^3+^-induced antioxidant response, cytotoxicity, and apoptosis, as well as the interplay between NRF1 and NRF2, in response to iAs^3+^ exposure, using HaCaT cells, a human keratinocyte cell line that models the skin as a target of iAs. In this study, we found direct evidence that iAs^3+^ activates both the NRF1- and NRF2-mediated antioxidant responses, which protects the cells from acute arsenic cytotoxicity. This indicates for the first time that NRF1 is a novel target of iAs^3+^ exposure. The results of this study provide important insights into the initial molecular response to iAs^3+^ in the target cells of arsenic toxicity and carcinogenicity.

## Materials and Methods

### Reagents and cell culture

We purchased sodium arsenite, sulforaphane (SFN), and *tert*-butylhydroquinone (tBHQ) from Sigma Chemical Co. (St. Louis, MO, USA) and tunicamycin (TU), thapsigargin (TG), and brefeldin A (BFA) from Calbiochem (San Diego, CA, USA). HaCaT cells, a spontaneously immortalized human epithelial cell line developed by Boukamp et al. (1988) were obtained from N.E. Fusening, German Cancer Research Center, Heidelberg, Germany. The cells were cultured in Dulbecco’s modified Eagle’s medium supplemented with 10% fetal bovine serum, 100 U penicillin/mL, and 100 μg streptomycin/mL, as previously described (Pi et al. 2003). Cultures were maintained at 37°C in a humidified 5% CO_2_ atmosphere. Culture media, fetal bovine serum, and supplements were purchased from Invitrogen (Carlsbad, CA, USA). The stock solutions of chemicals used in the current study were prepared in culture medium or 0.5% dimethyl sulfoxide (DMSO) in medium (vehicle).

### Lentiviral-based short-hairpin RNA (shRNA) transduction

We obtained MISSION shRNA lentiviral particles from Sigma. Lentiviral transduction of HaCaT cells with particles for shRNAs targeting NRF1 (SHVRS-NM_003204) or scrambled nontarget negative control (sh-Scr; SHC002V) was performed as described previously (Woods et al. 2009). The cells were maintained in medium containing 1.0 μg/mL puromycin.

### Quantitative real-time reverse-transcriptase polymerase chain reaction (RT–PCR) analysis

Total RNA was isolated with TRIzol (Invitrogen) and then subjected to cleanup using the RNase-Free DNase Set and RNeasy Mini Kit (Qiagen, Valencia, CA, USA). Quantitative real-time RT-PCR was performed as described previously (Woods et al. 2009). The primers {*NRF1* [GenBank accession no. NM_003204 (National Center for Biotechnology Information. 2010)], *HMOX1* (heme oxygenase 1; NM_002133), NQO1 (NM_000903), *SRX* (sulfiredoxin 1; NM_080725), *GCLC* (γ-glutamate cysteine ligase catalytic subunit; NM_001498), and *GCLM* (γ-glutamate cysteine ligase regulatory subunit; NM_002061), described in Supplemental Material, Table 1 (doi:10.1289/ehp.1002304)} were designed using Primer Express 3 (Applied Biosystems, Carlsbad, CA, USA) and synthesized by MWG-Biotech Inc. (High Point, NC, USA). Real-time fluorescence detection was carried out using an ABI PRISM 7900 Sequence Detector (Applied Biosystems).

### Western blot analysis

Isolation of cell fractions and Western blotting were performed as described previously (Pi et al. 2003; Woods et al. 2009). Briefly, whole-cell lysates (50 μg protein) were separated on 4–12% Tris-glycine gels. Antibodies for NRF1 (H-285) (sc-13031; 1:500), NRF2 (sc-13032; 1:500), and Kelch-like ECH-associated protein 1 (KEAP1; sc-15246; 1:500) were from Santa Cruz Biotechnology Inc. (Santa Cruz, CA, USA). Antibodies for GRP78/BIP (glucose-regulated protein, 78 kDa/binding immunoglobulin protein; no. 3177; 1:1,000) and CHOP (CCAAT/enhancer-binding protein homologous protein; no. 2895; 1:1,000), both downstream protein markers for ER stress, were purchased from Cell Signaling Technology, Inc. (Danvers, MA, USA). Antibodies for lamin A (L1293; 1:2,500), β-actin (A1978; 1:2,000), and α-tubulin (T5168; 1:2,000) were purchased from Sigma. Antibody for GCLC (RB-1697; 1:800) was obtained from Lab Vision (Fremont, CA, USA).

### Chromatin immunoprecipitation assay

We performed ChIP analyses using the EZ ChIP kit (Upstate Biotechnology, Temecula, CA, USA) according to the manufacture’s protocol. PCR amplification was carried out for 40 cycles with 5 μL of sample DNA solution, and PCR products were separated on 3% agarose gels in Tris–acetate–EDTA buffer. Two primers were used to amplify the segment flanking an active ARE site on NAD(P)H:quinone oxidoreductase 1 (*NQO1*) promoter with forward primer 5′-attacctgccttgaggagca-3′ and reverse primer 5′-cggattactgtggtgcccta-3′, which generate a 206-bp product.

### Acute cytotoxicity assay

A minimum of five replicates of 10,000 cells/well were plated in 96-well plates and allowed to adhere to the plate for 24 hr, at which time the medium was removed and replaced with fresh serum-free medium containing arsenic compounds. Cells were then incubated for an additional 24 hr, and cell viability was determined using the CellTiter Non-Radioactive Cell-Proliferation Assay Kit with MTT [3-(4,5-dimethylthiazol-2-yl)-2,5-diphenyltetrazolium bromide] (Promega, Madison, WI, USA). Measurements are expressed as a percentage of the untreated control of corresponding cells. The LC_50_ (concentration lethal to 50% of cells) values were determined from analysis of the log-linear phase of the curves.

### Determination of apoptosis by flow cytometry

Cells were seeded in a six-well plate and grown to approximately 80% confluence. After 20 hr of iAs exposure, the floating and attached cells were harvested for apoptosis analysis. We detected phosphatidylserine on the outer leaflet of apoptotic cells using the TACS Annexin V-FITC (fluorescein isothiocyanate) Apoptosis Detection Kit (Trevigen, Gaithersburg, MD, USA) as described previously (Pi et al. 2005). For each sample, 10,000 cells were examined by flow cytometry (Becton Dickinson FACSVantage; BD Biosciences, San Jose, CA, USA). We determined the percentage of apoptotic cells by statistical analysis of the various dot plots using CellQuest software (BD Biosciences).

### ARE reporter assay

We obtained Cignal Lenti ARE reporter, which expresses a luciferase gene driven by multiple ARE (TCACAGTGACTCAGCAAAATT) repeats, from SABiosciences (Frederick, MD, USA). Lentiviral transduction of HaCaT cells was performed as described previously (Woods et al. 2009). Cells were grown to approximately 90% confluency and subcultured in medium containing 1.0 μg/mL puromycin. The luciferase activity was measured by Luciferase Reporter Assay System (Promega) according to the manufacturer’s protocol. The luciferase activity was normalized to cell viability that was determined using the Non-Radioactive Cell-Proliferation Assay Kit (Promega).

### Statistical analyses

We performed all statistical analyses using GraphPad Prism, version 5 (GraphPad Software, San Diego, CA, USA), with *p* < 0.05 taken as significant. More specific indices of statistical significance are indicated in individual figure legends. Data are expressed as mean ± SE. For comparisons among groups, we performed one-way analysis of variance with Bonferroni post hoc testing.

## Results

### iAs^3+^ increases nuclear NRF1 accumulation

**Figure 1:**
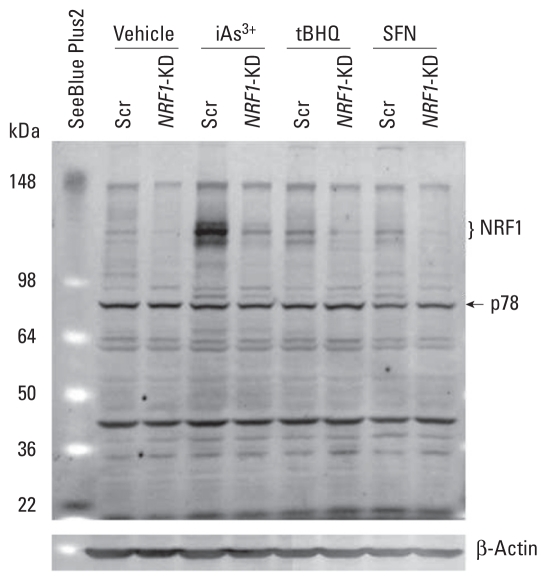
Representative image of NRF1 immunoblots with whole-cell lysates derived from *NRF1*-KD and Scr control HaCaT cells. Cells were treated with vehicle (medium), 20 μM iAs^3+^, 50 μM tBHQ, or 7.5 μM SFN for 6 hr. Whole-cell lysates (50 μg protein) were separated on 4–12% Tris-glycine gels and detected using anti-NRF1. β-Actin was used as a loading control, and SeeBlue Plus2 (Invitrogen) was used as an MW marker.

Based on the Ensembl database (Wellcome Trust Sanger Institute/European Bioinformatics Institute 2010), the human *NRF1* gene contains six exons, transcribes three splice variants, and translates into three proteins, NRF1-1, NRF1-2, and NRF1-3, with 742, 772, and 791 amino acids, respectively [see Supplemental Material, Table 2 (doi:10.1289/ehp.1002304)]. The predicted molecular weights (MWs) of NRF1-1, NRF1-2, and NRF1-3 are 81.5, 84.7, and 86.9 kDa, respectively. However, our immunoblots (Figure 1), using an antibody developed against an epitope corresponding to amino acids 191–475 mapping near the N-terminus of human NRF1, showed that multiple bands with apparent MWs approximately 120–140 kDa were dramatically diminished by knockdown (KD) of *NRF1* using lentiviral shRNA targeting human *NRF1* in HaCaT cells (*NRF1*-KD), suggesting that these immunoreactive bands represent endogenous human NRF1. Interestingly, these NRF1 protein bands significantly increased in response to iAs^3+^ but only marginally responded to tBHQ and SFN exposure. In addition, in response to iAs^3+^ treatment, a 78-kDa protein exhibited a pattern similar to that of the bands at 120–140 kDa (Figure 1). However, this protein was not detectable in nuclear fractions (data not shown), suggesting that this protein, if it represents an isoform of NRF1, is not associated with NRF1 transcriptional activity. Although we also observed multiple bands between 22 and 78 kDa on the blot, these bands lack correspondence to NRF1 silencing and were unaltered by iAs^3+^ exposure, suggesting that they may represent nonspecific binding of the antibody used for Western blot analysis. A 65-kDa isoform of mouse NRF1 has previously been identified and shown to potentially function as a dominant negative inhibitor of ARE-mediated transcription (Wang et al. 2007).

**Figure 2:**
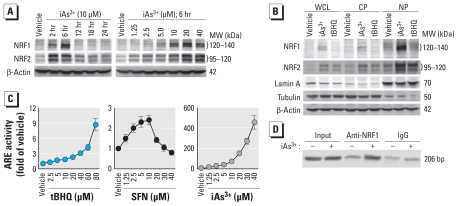
Acute iAs^3+^ exposure induces NRF1 accumulation and activates ARE-driven transcription in HaCaT cells. (*A*) iAs^3+^ time- and dose-dependently enhances NRF1 protein levels in whole-cell lysates compared with vehicle (medium). (*B*) iAs^3+^-induced NRF1 accumulates in nuclear fractions. Whole-cell lysate (WCL), cytosolic protein (CP), and nuclear protein (NP) cell fractions were collected after treatment with vehicle (medium), 10 μM iAs^3+^, or 25 μM tBHQ for 6 hr. (*C*) ARE-luciferase reporter in HaCaT cells is responsive to NRF2 and NRF1 activators, as shown in cells treated with vehicle (medium), tBHQ, SFN, or iAs^3+^ for 6 hr. Values shown are mean ± SE. (*D*) ChIP assay targeting an active ARE site in the *NQO1* promoter in HaCaT cells treated with 20 μM iAs^3+^ for 6 hr.

To investigate the involvement of NRF1 in iAs^3+^-induced antioxidant response in human keratinocytes, we measured the dose response and time course of iAs^3+^-induced NRF1 accumulation. As shown in Figure 2A, exposure to iAs^3+^ resulted in NRF1 protein accumulation in HaCaT cells in a time- and dose-dependent fashion that reached a peak at 6 hr. Consistent with our previous study (Pi et al. 2003), the same iAs^3+^ treatment also concomitantly induced NRF2 protein accumulation in a pattern similar to that of NRF1 (Figure 2A). Because nuclear accumulation is essential for a nuclear factor’s transcriptional activity, we determined the levels of NRF1 and NRF2 in subcellular fractions after iAs^3+^ exposure. We detected iAs^3+^-induced NRF1 and NRF2 mainly in nuclear fractions (Figure 2B), suggesting that NRF1 functions as a transcription factor, as does NRF2, in response to iAs^3+^ exposure. To determine the transcriptional activity of NRF1 and NRF2, we assessed the activity of the Cignal Lenti ARE reporter, which is designed to monitor the activity of the antioxidant response signal transduction pathway in cultured cells. HaCaT cells stably transduced with the ARE reporter showed a dose- and time-dependent induction of luciferase activity after tBHQ and SFN treatment, confirming that the cells are responsive to ARE activation [Figure 2C; see also Supplemental Material, Figure 1 (doi:10.1289/ehp.1002304)]. Consistent with the finding that iAs^3+^ strongly induced nuclear accumulation of both NRF1 and NRF2 (Figure 2A,B) but tBHQ and SFN are weak inducers of NRF1 (Figures 1 and 2B), we found iAs^3+^ to be a more potent activator for the ARE reporter than tBHQ and SFN (Figure 2C; see also Supplemental Material, Figure 1). To further confirm that NRF1 can bind to ARE, we performed a ChIP assay targeting an active ARE site on *NQO1* promoter (Dhakshinamoorthy and Jaiswal 2000). As shown in Figure 2D, acute iAs^3+^ exposure increased the binding of NRF1 with the ARE site of *NQO1* promoter.

### Effect of ER stressors on NRF1 protein modification

**Figure 3:**
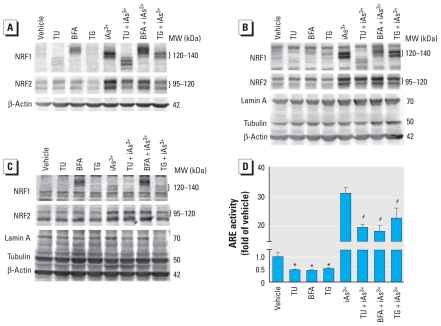
Effects of ER stressors on endogenous NRF1 migration on SDS-PAGE and nuclear accumulation. Immunoblots of whole-cell lysates (*A*), nuclear fractions (*B*), and cytosolic fractions (*C*) of HaCaT cells treated with vehicle (0.5% DMSO), 2 μg/mL TU, 1 μg/mL BFA, or 2 μM TG with or without 10 μM iAs^3+^ for 6 hr. (*D*) ER stressors reduced ARE luciferase activity induced by iAs^3+^. Values shown are mean ± SE. *p < 0.05 compared with vehicle. ^#^*p* < 0.05 compared with iAs^3+^ alone.

Previous studies have reported that NRF1 is a glycosylated protein sequestered in the ER and that ER stressors, including TU, BFA, and TG, have been found to affect the glycosylation status of recombinant human or murine NRF1 (Wang and Chan 2006; Zhang et al. 2009b). To study whether endogenous human NRF1 is regulated by the same mechanism, we investigated the effect of ER stressors on the migration of iAs^3+^-induced NRF1 using SDS-PAGE. As shown in Figure 3A, treatment of HaCaT cells with TU, an inhibitor of *N*-linked protein glycosylation (Shang et al. 2002), resulted in a faster migration of NRF1 proteins. In contrast, BFA, which blocks protein transport from the ER to Golgi (Li et al. 2006), led to accumulation of slower migrating NRF1, whereas TG, which blocks ER uptake of calcium by inhibiting sarcoplasmic/endoplasmic Ca^2+^-ATPase (Thastrup et al. 1990), did not affect NRF1 migration but slightly decreased iAs^3+^-induced NRF1 accumulation. To evaluate the effects of NRF1 modification by ER stressors on its transcriptional activity, we assessed nuclear NRF1 accumulation and ARE-reporter activity in HaCaT cells exposed to iAs^3+^ with TU, BFA, or TG. We observed the 120–140 kDa forms of NRF1 mainly in nuclear fractions (Figure 3B), suggesting that these forms may retain transcriptional activity. In contrast, we detected the slower migrating NRF1 induced by BFA mostly in cytosolic fractions (Figure 3C). Although TU + iAs^3+^–induced faster-migrating forms of NRF1 were detectable in nuclear fractions, the levels of these forms were much lower than those of the 120–140 kDa forms in nuclear fractions induced by iAs^3+^ alone or by TG + iAs^3+^ (Figure 3B). Consistent with the findings in immunoblots, all three ER stressors, which induced ER stress response at the concentrations used [see Supplemental Material, Figure 2 (doi:10.1289/ehp.1002304)], significantly reduced basal and iAs^3+^-induced ARE-reporter activity (Figure 3D).

Because NRF2 is another important transcription factor for ARE activation (Pi et al. 2003), we determined the effect of ER stressors on NRF2 expression. In contrast to the varied effects on NRF1, the three ER stressors had no obvious effect on NRF2 migration on SDS-PAGE (Figure 3A–C), suggesting that no protein modification occurred in NRF2. However, TU and BFA slightly enhanced basal NRF2 protein level, whereas TG decreased it (Figure 3A). Under iAs^3+^-exposed conditions, TU and TG obviously reduced NRF2 levels in whole-cell lysates and nuclear fractions, whereas BFA had little effect (Figure 3B).

### Involvement of NRF1 in iAs^3+^-induced antioxidant response

To study the role of NRF1 in iAs^3+^-induced antioxidant response and cytotoxicity, we performed lentiviral shRNA-mediated knockdown of *NRF1* in HaCaT cells, using five shRNAs against *NRF1* for transduction [see Supplemental Material, Table 3 and Figure 3 (doi:10.1289/ehp.1002304)]. One of the constructs (sh-*NRF1-5*) markedly silenced *NRF1* expression compared with Scr (sh-Scr), whereas the other four constructs had a moderate silencing effect. Because the level of NRF1 protein is barely detectable in untreated cells and even in tBHQ- or SFN-challenged cells (Figure 1), the efficiency of knockdown by sh-*NRF1-5* (*NRF1*-KD cells) was confirmed by notably diminished induction of NRF1 caused by iAs^3+^ exposure (Figure 1; see also Supplemental Material, Figure 3B). Furthermore, the expression of NRF1 downstream targets GCLC and GCLM were attenuated (see Supplemental Material, Figure 3C,D), indicating that NRF1 activity is suppressed in *NRF1*-KD cells. Along with the reduction of GCLC and GCLM, the intracellular GSH level was significantly reduced by silence of *NRF1* (see Supplemental Material, Figure 3E), confirming that NRF1 is critical in regulation of GSH synthesis.

**Figure 4:**
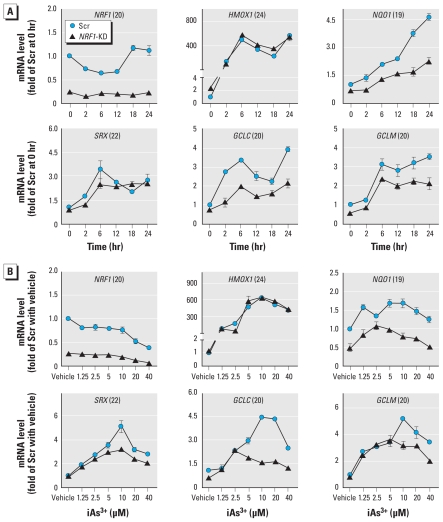
NRF1 regulates some ARE-dependent genes induced by iAs^3+^ in HaCaT cells. (*A*) Time course of ARE-dependent gene expression induced by 10 μM iAs^3+^. (*B*) Dose response of iAs^3+^-induced ARE-dependent gene expression; cells were exposed to vehicle (medium) or iAs^3+^ for 6 hr. The number in parentheses after each gene name is the Ct (cross threshold) value of that gene in Scr cells.

**Figure 5:**
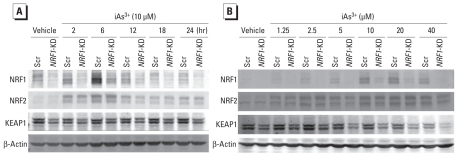
Effect of iAs^3+^ on protein expression of NRF1, NRF2, and KEAP1 in Scr and *NRF1*-KD cells. HaCaT cells were exposed to vehicle (medium) or 10 μM iAs^3+^ for indicated times (*A*) or to vehicle (medium) or indicated concentrations of iAs^3+^ for 6 hr (*B*).

To define the molecular basis for how NRF1 is involved in cellular oxidative defense against acute iAs^3+^ toxicity, *NRF1*-KD and Scr cells were acutely exposed to iAs^3+^; we then determined the inducible expression of ARE-dependent genes, including *HMOX1*, *NQO1*, *SRX*, *GCLC*, *GCLM*, and *NRF1*, at mRNA (Figure 4) and protein levels [see Supplemental Material, Figure 3 (doi:10.1289/ehp.1002304)]. In Scr cells, iAs^3+^ dose- and time-dependently increased NRF1 protein levels (Figure 5) and enhanced the mRNA levels of ARE-dependent genes (Figure 4). Knockdown of *NRF1* substantially decreased NRF1 accumulation (Figure 5) and the expression of *NQO1*, *GCLC*, and *GCLM* under basal and iAs^3+^-exposed conditions (Figure 4). Interestingly, induction of *HMOX1* caused by iAs^3+^ did not depend on NRF1 (Figure 4).

A previous study (Leung et al. 2003) revealed that NRF1 and NRF2 have overlapping roles in regulating basal expression of ARE-dependent genes. Thus, we studied the cross talk of NRF1 with NRF2, as well as with KEAP1, a well-known negative regulator of NRF2 transcriptional activity (Hayes and McMahon 2009). As shown in Figure 5, silencing of *NRF1* in HaCaT cells did not disturb iAs^3+^-induced NRF2 accumulation. However, lack of *NRF1* decreased protein levels of KEAP1 under basal and iAs^3+^-challenged conditions, although KEAP1 was not affected by iAs^3+^ treatment.

### iAs^3+^-induced cytotoxicity and apoptosis in NRF1-deficient HaCaT cells

**Figure 6:**
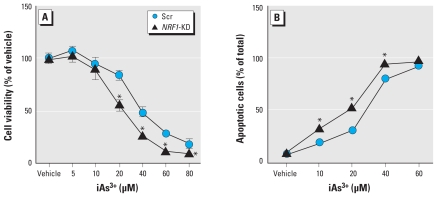
*NRF1*-KD cells are sensitized to iAs^3+^ cytotoxicity. (*A*) Cell viability in response to 24-hr iAs^3+^ exposure measured by MTT (*n* = 6). (*B*) Quantification of iAs^3+^-induced apoptosis determined by flow cytometry. Cells were exposed to iAs^3+^ for 20 hr; annexin V–positive cells were quantified as apoptosis cells (*n* = 3). *p < 0.05 compared with Scr cells exposed to the same concentration of iAs^3+^.

To investigate the roles of NRF1 in iAs^3+^-induced cytotoxicity, we measured the acute (24 hr) effect of iAs^3+^ on cell metabolic integrity in *NRF1*-KD cells. Selective deficiency of *NRF1* in HaCaTs significantly enhanced the sensitivity to iAs^3+^ toxicity (Figure 6A). The LC_50_ value (mean ± SE) was 28.62 ± 3.06 μM in *NRF1*-KD cells, whereas it was 35.99 ± 2.11 μM in Scr cells. To further substantiate these findings, we measured iAs^3+^-induced apoptosis and necrosis using flow cytometry with Annexin V-FITC and propidium iodide double staining. Consistent with the results of cytotoxicity, the knockdown of *NRF1* in HaCaT cells significantly enhanced the sensitivity to iAs^3+^-induced apoptosis [Figure 6B; see also Supplemental Material, Figure 4 (doi:10.1289/ehp.1002304)].

## Discussion

NRF1 is a ubiquitously expressed transcription factor that occurs in a wide range of tissues (Biswas and Chan 2010; Luna et al. 1994). Skin is a major target organ for the chronic toxic and carcinogenic effects of iAs (Yoshida et al. 2004). Our previous studies revealed that chronic induction of ARE-dependent genes may be linked to acquired apoptotic resistance and malignant transformation of keratinocytes following iAs^3+^ exposure, whereas NRF2 has been recognized as a key transcription factor in iAs^3+^-induced antioxidant response (Pi et al. 2003, 2007, 2008). The present study provides the first demonstration that long isoforms (120–140 kDa) of NRF1 also contribute to iAs^3+^-induced antioxidant response in human keratinocytes and suggests that activation of NRF1 is potentially involved in chronic dermal arsenic toxicity.

Hyperkeratosis and cancer are the most common human skin disorders caused by chronic iAs exposure (IARC 1987; Pi et al. 2000; Wong et al. 1998; Yoshida et al. 2004). However, the underlying mechanism is unclear. It has been reported that disruption of *Keap1* in mice leads to skin hyperkeratosis, most likely because of constitutive activation of NRF2 and aberrant expression of some ARE-dependent cytokeratins (Itoh et al. 2003). In humans, increased expression of ARE-dependent genes, resulting from mutations in *KEAP1* and/or *NRF2*, has been linked to a malignant phenotype in the lung and other organs (Kwak and Kensler 2009. Padmanabhan et al. 2006; Shibata et al. 2008a, 2008b; Singh et al. 2006; Stacy et al. 2006). Given the importance of NRF1 (Figure 4) and NRF2 (Pi et al. 2003) in regulating the expression of ARE-dependent genes induced by iAs^3+^, it is highly possible that NRF1 and/or NRF2 activation plays a pathogenic role in skin disorders chronically induced by arsenic exposure, including carcinogenesis, although additional research is required to confirm this.

Apoptosis normally functions to control the integrity of cell populations by eliminating aberrant clones, whereas failure of apoptosis likely is a key contributor to tumor initiation and progression, as well as drug resistance in skin cancer and cancer in general (Guzman et al. 2003; Hanahan and Weinberg 2000). Thus, an acquired, generalized apoptotic resistance is an important event in the process of arsenic-induced malignant transformation (Pi et al. 2008). Our previous data indicated that HaCaT cells chronically treated with iAs^3+^ show a generalized resistance to apoptosis and malignant transformation, which may be associated with enhanced basal NRF2 activity (Pi et al. 2005, 2008). Here, for the first time we report that NRF1 also contributes to iAs^3+^-induced ARE-dependent gene expression and protects cells from acute arsenic toxicity, suggesting that NRF1 may be another key transcription factor in arsenic carcinogenesis. However, whether NRF1 activation is involved in acquired apoptotic resistance in malignant transformation induced by chronic iAs^3+^ exposure needs further study.

It has been predicted that human *NRF1* gene may transcribe at least four different transcripts with alterative first exons, differential splicing, and alterative polyadenylation (Biswas and Chan 2010). In addition to the long isoforms as we observed in HaCaT cells, a 65-kDa isoform of mouse NRF1 has been identified and shown to potentially function as a dominant negative inhibitor of ARE-mediated transcription (Wang et al. 2007). Although we observed two bands close to 65 kDa on Western blots in the present study (Figure 1), neither corresponded to *NRF1* silencing or were altered by iAs^3+^ exposure, suggesting that they may represent nonspecific binding of the antibody used for analysis. This discrepancy, which could be due to differences in cell types, treatment, and antibodies used for immunoblotting, needs further study.

Previous studies have suggested that NRF1 is sequestered in ER and that oxidative stress activates NRF1 by permitting accumulation into the nucleus (Biswas and Chan 2010). The ER is a central organelle as the place of lipid synthesis, protein folding, and protein maturation (Banhegyi et al. 2007). As a major intracellular calcium storage compartment, the ER also plays a critical role in maintenance of cellular calcium homeostasis (Li et al. 2006). ER stress (conditions interfering with the function of ER) can be induced by accumulation of unfolded proteins and excessive protein traffic (Banhegyi et al. 2007; Li et al. 2006). ER stress could also be elicited in the cell culture system by pharmacological agents, including TU, BFA, and TG, through distinct molecular mechanisms (Li et al. 2006; Shang et al. 2002; Thastrup et al. 1990). Consistent with previous studies using recombinant human or murine NRF1 (Wang and Chan 2006; Zhang et al. 2009b), treatment of HaCaT cells with TU, an inhibitor of *N*-linked protein glycosylation (Shang et al. 2002), resulted in faster migration of NRF1 isoforms on SDS-PAGE, suggesting that long isoforms of endogenous human NRF1 are glycosylated proteins. In contrast, BFA, which blocks protein transport from ER to Golgi (Li et al. 2006), led to accumulation of slower migrating NRF1 proteins, suggesting that NRF1 may be further glycosylated in ER if its transportation to Golgi is blocked. TG, which blocks ER uptake of calcium by inhibiting sarcoplasmic/endoplasmic Ca^2+^-ATPase (Thastrup et al. 1990), slightly decreased iAs^3+^-induced NRF1 accumulation but did not affect migration on SDS-PAGE. The finding that ER stressors TU, BFA, and TG affect NRF1 migration on SDS-PAGE differently suggests that ER stress may not be a common mechanism for NRF1 modification. Although ER is an important organelle for NRF1 posttranslational modification and may be involved in NRF1-mediated antioxidant response, the molecular basis for how ER participates in NRF1 activation needs further investigation.

Biswas and Chan (2010) have reported that NRF1 and NRF2 have overlapping roles in regulating basal expression of ARE-dependent genes. In the present study we found that basal and inducible expression of some ARE-driven genes, such as *GCLC*, *GCLM*, and *NQO1*, are highly dependent on NRF1. However, the induction of HMOX1 by high concentrations of iAs^3+^ was independent of NRF1, suggesting that HMOX1 is not regulated by NRF1. It should be noted that NRF1/NRF2-independent mechanisms for iAs^3+^-induced expression of GCLC and GCLM have been demonstrated in murine hepatocytes and mouse embryo fibroblasts (Thompson et al. 2009). This inconsistency with the present study suggests that forms of human and mouse NRF1 behave differently or, more likely, reflects differences between the cell types evaluated in *in vitro* assays. As with NRF2, NRF1 has been postulated to interact with KEAP1 (Biswas and Chan 2010), although the biological significance of this reaction is poorly characterized. In the present work, lack of NRF1 in HaCaT cells did not disturb iAs^3+^-induced NRF2 accumulation but noticeably decreased KEAP1 protein levels under basal and iAs^3+^-exposed conditions, suggesting a potential interaction between NRF1 and KEAP1. If KEAP1 could serve as a negative regulator of NRF1, decreased KEAP1 expression caused by NRF1 silencing may represent a compensation mechanism to maintain the overall cellular ARE activity. However, this hypothesis needs further investigation.

In the present study, we found convincing evidence that NRF1 is involved in the regulation of the ARE gene battery induced by iAs^3+^ and contributes to the resistance against iAs^3+^-induced cytotoxicity and apoptosis. Importantly, we demonstrated arsenic activation of NRF1 in a human skin cell line, implicating an NRF1-mediated oxidative stress response cascade as an important event in a potential target cell of arsenic carcinogenesis. Given the potential importance of oxidative stress in arsenic dermal toxicity and carcinogenicity, as well as the critical role of NRF1 in the defense against oxidative damage, our findings provide an important insight into the mechanism of chronic arsenic dermal toxicity.
